# Mixed-methods approach in evaluating safe abortion care services at public health facilities in North Shewa zone, central Ethiopia: a multicenter institutional cross-sectional study

**DOI:** 10.3389/frhs.2024.1352178

**Published:** 2024-07-05

**Authors:** Aklilu Tamire, Bezawit Birhanu, Abraham Negash, Mesay Dechasa, Awoke Masrie, Samrawit Shawel, Jerman Dereje, Tilaye Gebru, Obsan Kassa Tafesse, Dechasa Adare Mengistu, Addisu Sertsu, Dawit Wolde Daka

**Affiliations:** ^1^ School of Public Health, College of Health and Medical Science, Haramaya University, Harar, Ethiopia; ^2^ Faculty of Public Health, Department of Health Policy and Management, Jimma University, Jimma, Ethiopia; ^3^ School of Nursing and Midwifery, College of Health and Medical Sciences, Haramaya University, Harar, Ethiopia; ^4^ Department of Clinical Pharmacy, School of Pharmacy, College of Health and Medical Science, Haramaya University, Harar, Ethiopia; ^5^ Department of Psychiatry, School of Nursing and Midwifery, College of Health and Medical Sciences, Haramaya University, Harar, Ethiopia; ^6^ Department of Environmental Health, College of Health and Medical Science, Haramaya University, Harar, Ethiopia

**Keywords:** evaluating, mixed methods, safe abortion, service, care, North Shewa

## Abstract

**Background:**

Of the 55.7 million abortions that were performed globally, 25.1 million (45.1%) were not safe. Nearly 97% of these took place in developing countries. Approximately 71% of economically developed countries allow safe abortion care (SAC) services, whereas only 16% of developing countries permit it. In sub-Saharan Africa, 92% of mothers live in 43 countries where SAC services are restricted by law. Most Ethiopian women continue to self-terminate unwanted pregnancies in hazardous conditions. The aim of this evaluation was to assess input, care providers’ compliance with national guidelines, and clients’ satisfaction.

**Methods:**

A multicenter cross-sectional study design with a mixed-methods approach was used. Seven public health facilities were randomly selected where 75 health caseworkers were directly observed; 296 clients and 14 key informants were interviewed, respectively. A resource inventory checklist was used to assess all inputs. The overall SAC services evaluation was summarized from 40 indicators: 13 resource availability indicators, 14 healthcare workers' compliance to national guidelines indicators, and 13 clients' satisfaction toward SAC services indicators. A multivariate logistic regression model was fit to determine factors that affect client satisfaction at a *p*-value <0.005.

**Results:**

There were 75 healthcare providers in the maternal and child health departments in the study area. Except for the interruption of water and electricity, maternal waiting area, counseling, and procedural room, all are available making 94% of resources availability. All healthcare workers were compliant in providing anti-pain medication during procedures, identifying clients if they were targeted for an HIV/AIDS test, and providing their test results as per the guideline. Nevertheless, they were poorly compliant in respecting the clients (9, 12%) and taking vital sign (33, 44%). The overall compliance was 62.3%, while only 51% were satisfied with waiting time and privacy of counseling room. The overall client satisfaction was 65%. The overall evaluation of SAC services was 72.9%.

**Conclusion:**

Resource availability was excellent, which was in line with national SAC expectations while the healthcare workers’ compliance to national guidelines was fair, which deviated from expectations. The clients’ satisfaction and the overall evaluation were good, which was below the hypothesized expectation.

## Introduction

Safe abortion care (SAC) is defined as a procedure carried out by the expertise of healthcare workers using medication and surgery as per World Health Organization (WHO) recommendations and an appropriate duration of pregnancy under hygienic conditions (1). It is a program proposed to reverse the prevalence of unsafe abortions and the complications that arise from them. Globally, there have been 55.7 million abortions, from which 25.1 million (45.1%) were unsafe; of them, 97% occurred in developing countries (1). Only 16% of developing countries allow safe abortion care services by law whereas approximately 71% of developed countries allow it in their constitution (2, 3). It is estimated that 30, 220, and 520 maternal deaths per 100,000 are due to risky abortions in developed, developing, and sub-Saharan Africa (SSA), respectively (4). The problem of unsafe abortion and its related maternal death rate are disproportionately higher in Africa than other continents (5). As a result of unsafe abortion, close to 7 million mothers are treated as inpatients every year in developing nations; of them, 4.7%–13.2% of maternal deaths are due to unsafe abortion (4). In 43 nations of SSA, 92% of women were not receiving SAC service as it is highly restricted according to the rules of the countries. Currently, some nations offer safe and facility-based SAC services after expanding the legal foundation for the service and adapting regulations from international medical standards. However, the majority of SSA countries have legislative restrictions, and stigma is causing women to undergo abortions less securely, painfully, and covertly (1).

In Ethiopia in 2005, maternal mortality was 687 per 100,000 live births (before the establishment of the national safe abortion care service), with complications from unsafe abortion being responsible for one-third of these deaths (6), while in Ethiopia, the combined rate of maternal deaths from unsafe abortions is currently 8.6% (7).

To reverse the high maternal morbidity and mortality resulting from the termination of an unhealthy pregnancy, the Federal Ministry of Health (FMOH) drafted practical and procedural guidelines for SAC services in 2006. In this manual certain criteria were included after the approval of the House of Representatives of People (HRP) under article 551 sub-article 1 of the penal codes of Ethiopia. After the first edition introduction of the SAC guideline by the FMOH, Ethiopia became a country that reduced maternal death significantly. Before the introduction of the SAC service guideline, the attribution of unsafe abortion to maternal death was approximately 32%, which was far less than the current prevalence of 6%–9%. According to the Ethiopian Health and Demographic Health Survey (EDHS) 2016, maternal mortality was reduced to 412/100,000. This is after the full implementation of the national SAC services guideline or 10 years after the SAC services guideline. The number of lives saved and complications averted was a substantial attainment (2). However, religion and healthcare workers’ attitudes are the main obstacle to implementing SAC services as intended. Although several care providers took the SAC services training, only one-quarter of healthcare workers agreed to perform SAC services while the rest refused to perform the services for religious and personal reasons (8). Usually, healthcare providers take SAC training only to benefit from the training payment and other incentives. On the other hand, only 10.4% of healthcare providers believed that SAC service legalization can prevent unsafe abortion (8). In addition, 70% of Ethiopian healthcare workers were not comfortable working a SAC service center because it was against their religious and personal values (9), which can heavily affect compliance with the guideline, which can further affect client satisfaction. Furthermore, stigma, discrimination, male domination, family pressure, poor structure (input)—especially in the remote areas—and poor referral systems were factors to undermine client satisfaction with SAC services (10).

Healthcare institutions of Ethiopia are facing a challenge to improve client satisfaction toward SAC services. Indeed, fulfilling the clients’ expectations has become a current concern and essential criterion in the assessment of healthcare facilities’ performance and public health programs (11). Factors that undermine client satisfaction were as follows: poor availability of healthcare service, insufficient equipment, and lack of trained staff. In addition, the low standard of facilities and the lack of abortion medication are factors that affect the quality of SAC services (12). Though improving clients’ satisfaction and healthcare workers’ compliance is an important intervention, the status of available resources and how compliant healthcare workers to standards were lacking evidence in most health facilities (13).

Despite the significant achievements of the SAC service coverage, still much remains in infrastructure (resources) availability and healthcare workers’ compliance with the national guideline and fulfilling clients’ expectations. In Ethiopia, unwanted pregnancies continue to be terminated in infectious conditions that may attribute to increased pregnancy complications for many reasons (14). Hence, the aim of the current study was to describe the availability of resources in terms of trained manpower, medications, supplies, infrastructure, and whether healthcare workers delivery services were in accordance with the national SAC service recommendations. Additionally, it aimed to determine clients’ expectations and satisfaction regarding all services they received, and exploring facilitator factors that undermine SAC services.

## Materials and methods

### Study setting and period

This study was done at public health facilities in North Shewa zone, one of the 21 zones of Oromia regional state in Ethiopia. Its estimated total population is more than 1.5 million, and the number of males and females were comparable. On the other hand, the number of fertile women is approximately 100,000 (9). Information from the zonal health office revealed that the zone has 5 hospitals, 34 health centers, and 62 health posts, and the annual abortion prevalence rate in the region is 28 per 1,000 (15). The evaluation period was between 1 May and 30 May 2021.

### Study design

A mixed-methods approach and multicenter cross-sectional study design were used. Methods of data collection were direct observation of interactions between clients and care providers, key informant interviews (KIIs), and client exit interviews.

### Study population

This study was conducted in 14 woredas of North Shewa zone of Oromia reginal state, Ethiopia, from where seven health facilities were selected using simple random sampling. Hence, the study population consisted of SAC clients with a gestational age less than 28 weeks, documents healthcare providers, all SAC service focal persons, heads from selected health facilities, and the zonal health department head. Clients, documents, and healthcare workers were sources of quantitative data while the rest were sources of qualitative data. Three hospitals and four health centers were selected using the lottery method of the simple random sampling method.

### Samples size determination

The sample size of client exit interviews for the client satisfaction dimension was determined using a single population proportion formula based on the following assumptions. Required sample size, $n=(Z\alpha /2{)}^{2}p\;(1-p)/d^{2}$ where, **Z***α*/2 = 1.96 [95% confidence interval (CI)], *p* = proportion of clients satisfied with SAC service (76.3%) (16), and *d* = 5% level of margin of error (*d* = 0.05); $n=\frac{{\left(1.96\right)}^{2}\times 0.76(1-0.24)}{{\left(0.05\right)}^{2}}=280$.

Since the source population was less than 10,000, which was last year's third quarter SAC achievements in the zone and revealed that 545 were taken as the source population, it should be reduced using the reduction formula $nf=\frac{no}{1+\frac{no-1}{N}}\;\;=\frac{280}{1+\frac{279-1}{601}}\;\;=\frac{280}{1+\frac{280-1}{545}}\;=\frac{280}{1.5}=187$.

By considering 1.5, the design effect was 1.5 × 187 = 281. To compensate for the non-respondent rate, by adding 10% of the total sample size, 309 clients were required. These clients were interviewed to answer their level of satisfaction regarding the following concepts: respectfulness of care provider; privacy, treatment of guard at entrance or gate; preoperative information given, general information about post-abortion complications, skill of care providers, pain control during procedure, post-abortion family planning (FP) advice, the time taken to receive SAC services, cleanliness of the abortion procedural room, having a say in the decision-making regarding their problem, general information given on reproductive health, and ease of receiving laboratory services.

On the other hand, all available healthcare workers were observed (*n* = 75). To observe the compliance of care providers with the SAC service national guideline, 32 direct observations were conducted, four per health facility in three hospitals and one health center. However, three direct observations were conducted in the remaining three health centers.

Furthermore, 14 key informants who received previous training on SAC service were selected specifically for KIIs based on their knowledge of SAC services and the duration of their current position (>6 months) in all sampled health facilities. Selecting KIIs was guided by the idea of data saturation and stopped as it was reached. The interview guide was used during KIIs.

### Sampling procedure

**Figure 1:**
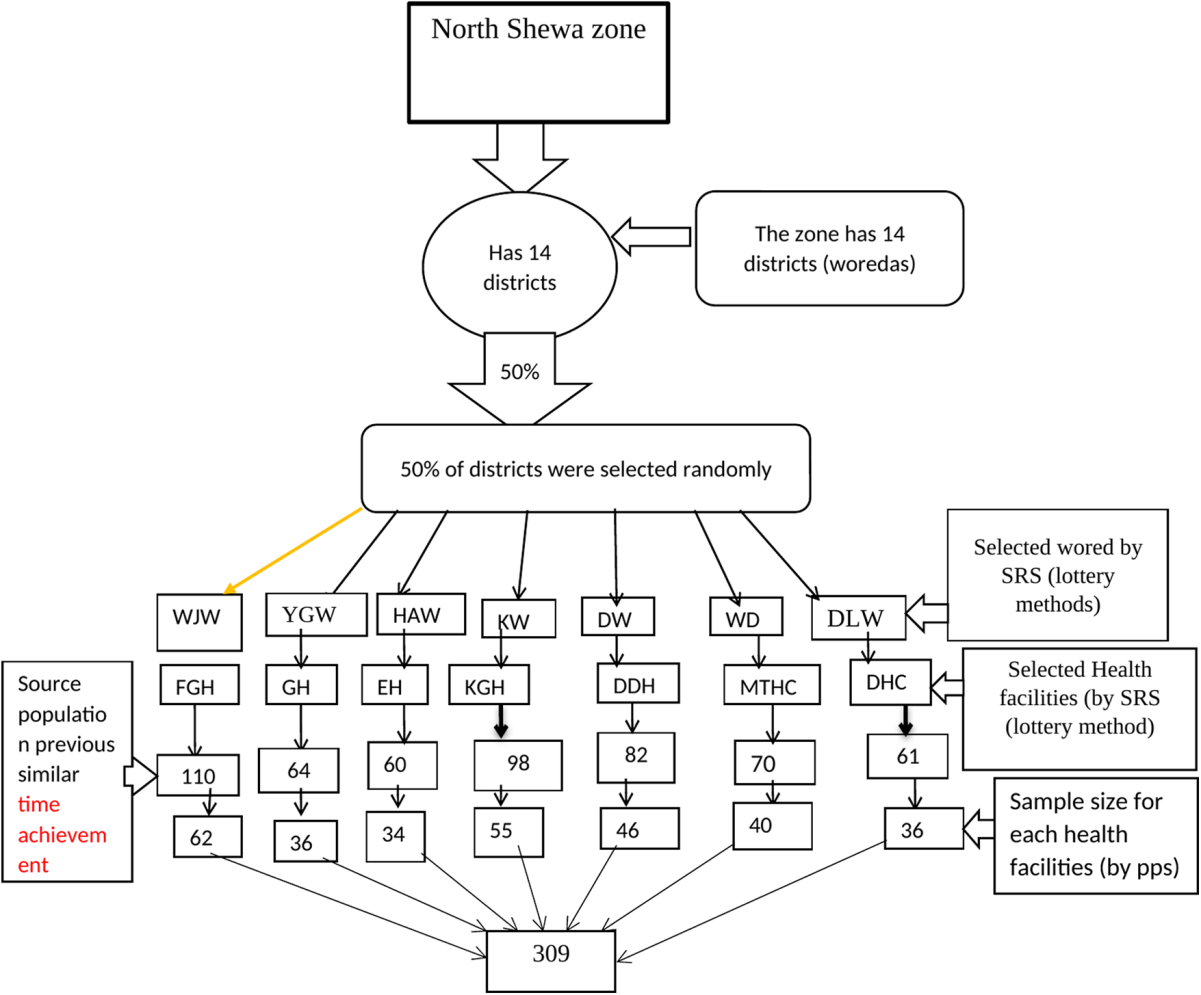
Proportional allocation of sample size for client exit interviews in each health facility at North Shewa zone government health facilities. KW, Kuyu Woreda; HAW, Hidabu Abote Woreda; DW, Dera Woreda; WJW, Wara Jarso Woreda; DLW, Debrlibanos Woreda; MTW, Muka Turi Woreda; KGH, Kuyu General Hospital; EH, Ejere Health Center; DDH, Dera District Hospital; FGH, Fitche General Hospital; DtsHC, Dabratsige Health Center; MTH, Muka Turi Health Center; YGHC, Yaya Gulalle woreda Fital Health Center.

All interviews regarding the structure/input needed to perform SAC services were assessed; in addition, resource managers were interviewed. Interactions between clients and care provider were observed 32 times (75 healthcare providers) in health facilities on days separate from the client exit interviews. The sample sizes for all facilities were allocated proportionally based on the clients’ caseload. The multistage sampling technique was considered to reduce the sampling error by adding a design effect of 1.5 to the client sample size (Figure 1). Finally, a consecutive interview was conducted for clients visiting the health facilities during the evaluation period.

### Studied indicators/variables

#### Resources availability indicators

Availability of the following resources was assessed: basic equipment, functional laboratory services, basic supplies, materials for infection prevention, equipment used for uterine evacuation, trained staff, technical and procedural guidelines, comprehensive abortion care (CAC) registration books, functional ambulance services, recording formats, procedural rooms, and maternal waiting area.

#### Healthcare worker compliance indicators

Healthcare worker compliance was assessed using the following indicators: respecting clients, explaining the steps of the procedure, taking gynecological history, doing a physical examination, taking vital signs before the procedure, providing contraceptives, providing anti-pain medication, providing post-abortion family planning counseling, declaring clients’ HIV/AIDS test status, assisting clients to express their desires, providing information supported by information education communication materials, wearing infection prevention materials, and sending baseline laboratory investigations.

#### Clients’ satisfaction indicators

Clients satisfaction was assessed using the following indicators: ease of getting laboratory service, pain management, clarity of information received, two-way communication during care provision, post-abortion family planning counseling, respectfulness of care providers, waiting time to receive the service, privacy of counseling room, treatment from the guards on entry and exit, cleanliness of safe abortion room, technical skills of care providers, information provided on safe abortion procedure, and information given on post-abortion complications.

#### Data collection tools development and procedures

In this evaluation, multiple data collection tools were used to obtain adequate data. The resource inventory checklist used includes human resources, instruments, laboratory services, basic supplies, drugs, recording, and the reporting format that was developed based on the national and WHO safe abortion care service guidelines (17, 18).

A direct observational checklist of interactions between clients and care providers was adapted from the International Pregnancy Advisory Services (IPAS) client-oriented and provider-efficient service for the CAC tool book (19) that has “yes” or “no” options whereas clients’ exit interview tools were developed from a similar previous study (16, 20). The tools were structured in to three parts. Part 1 consists of clients’ sociodemographic characteristic, part 2 consists of client- and service-related characteristics, and part 3 consists of client satisfaction. The tool has 13 items with a 5-point Likert scale in which satisfaction levels vary from 0 (very dissatisfied) to 5 (very satisfied). All parts of the client exit interview were translated into the local language and back to English for uniformity checking by language professionals. An evaluator developed an in-depth interview guide based on the aim of the evaluation by referring to national SAC service guidelines.

Seven data collectors with midwifery bachelor degrees were employed. A member of the maternity staff with a MSc degree and a midwife with a BSc degree supervised the overall data collection process. After oral informed consent was obtained from both the healthcare workers and the clients, interactions between the clients and healthcare providers were observed. The direct observer followed the health providers’ protocol, including wearing the uniform (gown) during observation. Permission letters for document reviews were obtained from the head of the zone's health department to each health facility. KIIs were conducted after direct observation. Field notes for each question and their responses were taken in the local language (Afan Oromo) and a tape recorder was used to capture their responses properly.

#### Data quality control

Training was given to supervisors and data collectors about the overall evaluation in relation to the overview of the study and data collection instruments so that the accuracy of data collection procedure could be maintained. Five percent of the sample size was used for a pretest on a similar target group.

The data collecting processes were overseen thoroughly and the day-to-day performance was checked immediately and scheduled for the subsequent day to solve any problem encountered. To ensure compliance with the data collecting protocol, the principal evaluator and supervisors check the collected data for completeness. The evaluation team (including the principal evaluator, overseers, and supervisors) and data gatherers conducted a meeting to discuss the challenges. The data collectors had developed a rapport with the healthcare workers being observed before a procedure so that they felt as comfortable as when they were not under observation. Healthcare providers and clients were informed that the data were required only for the improvement of SAC services and not for judgment on their performance.

#### Data analysis procedure

After checking the data quality, quantitative data were entered into EpiData version 4.6.2 (EpiData, Copenhagen, Denmark) and exported to SPSS version 25 (SPSS, CA, USA) for cleaning, editing, and coding. Descriptive statistics were used to present quantitative data in tables and figures. After the assumptions in linear regression analysis, including linearity, normality, multicollinearity, outlier, and homoscedasticity, were found to be fit, a multivariable logistic regression model was fit to assess the factors that affect client satisfaction. The general level of patient satisfaction was determined by the mean of the percentage of maximum scale score (PMSS) calculated using the formula $\mathrm{PMSS}=\frac{\mathrm{Actual}\;\mathrm{value}\;-\mathrm{Potential}\;\mathrm{minmum}\;}{\mathrm{Potential}\;\mathrm{maximum}-\mathrm{potential}\;\mathrm{minimum}}\times 100$ (21).

The association between the independent and dependent variables (client satisfaction) of this evaluation study was checked using a *p*-value <0.05% and a 95% confidence interval as the criterion for significance association.

A qualitative data analysis was performed manually after being transcribed into the original language and translated into English. The number of KIIs were guided by information and information saturation was reached with the participation of 14 key informants. The last transcription was checked against the notes to ensure quality of data. Before the analysis, the transcript was reread several times to fully understand the data. The various replies were compared depending on likenesses and organized into subthemes and themes. Quotes that represent many themes and articulated quotes said frequently by the majority of key informants were chosen. Under each dimension, indicators were used to judge the overall evaluation of SAC service.

## Results

### Resources availability

In this study, we assessed the availability of human resources, basic supplies, infrastructure, infection prevention, and laboratory service. This study revealed that 31 (70%) care providers in the MCH unit were trained for SAC services. These healthcare workers (HCW) were gynecologists, emergency surgeons, health officers, midwives, and nurses. Despite their distribution and adequacy, all health facilities had trained care providers. However, one health center had only one trained professional who also provides other additional maternal and child health services (Table 1). Most of the healthcare workers usually did not want to perform SAC procedures for religious and personal reasons, while others in certain health facilities found their overlapping duty as the SAC-trained individual were very limited. One of the participants emphasized, “Majority of the SAC trained care providers did not accept to deliver SAC services after took the SAC training due to their religion prohibition” (female maternal and child health program coordinator, 31).

**Table 1:** Availability of human resources and type of safe abortion care carried out at maternal and child health departments at North Shewa zone public health facilities.

Health profession	Health facilities															
	FGH		KGH		DDH		EHC		DtsHC		MTHC		YGHC		Total	
	U	T	U	T	U	T	U	T	U	T	U	T	U	T	U	T
Gynecologist	**	1	**	1	**	1	0	0	0	0	0	0	0	0	0	3
Emergency surgeon	*	0	*	2	*	2	*	0	*	0	*	0	*	0	*	4
Midwives	10	4	9	4	8	2	3	2	3	3	3	3	0	1	36	16
Nurses	0	0		0	1	0	0	0		0		0	0	1	1	1
Health officer	0	0	0	0	0	0	2	2	2	1	3	1	0		7	3
Total	10	5	9	7	9	5	5	4	5	4	6	4	0	2	44	31

FGH, Fiche General Hospital; KGH, Kuyu General Hospital; DDH, Dera District Hospital; EHC, Ejere Health Center; DtsHC, Dabratsige Health Center; MTHC, Muka Turi Health Center; YGHC, Yeya Gulale Health Center; U, untrained; T, trained.

*U (un trained) not represent Emergency surgeon.

**Gynecologist not need basic training for SAC services as he/she has advanced degree. U (un trained) not represent gynecologist.

Evidence from key informants shows that there were shortages of SAC-trained human resources in most health facilities. A key informant from one health center explained it as “Currently we faced SAC rained human resource shortage in MCH unit. We have only two SAC-trained professionals; one from the nurse department who is not active on SAC service because of workload in another nursing service” (man, age 35 years).

On the other hand, some healthcare providers refused to perform SAC services even after they took the training. That means they took the training for its incentives instead of for applying the training. One of the key informants said “Even if the trained profession is available, they don't accept to perform SAC service procedure by connecting it with religion prohibition which is a big obstacle to performing this service” (man, age 35 years).

### Availability of basic supplies, drugs, infrastructure, infection prevention, and laboratory services

All assessed health facilities (*n* = 7) have had basic supplies, drugs, infrastructure, infection prevention materials, and essential equipment in both the SAC services unit and in the storeroom to perform SAC services for the past 1 year. In addition, there were no health facilities with stockout of the following medications, including medication for abortion, anti-pain medicine, and antibiotics in the past year. However, some of the health facilities (Ejere, MukaTuri, and Yeya Gulalle health centers) had no waiting area and separate counseling and procedural rooms (Supplementary Table S1).

One of the key informants said, “We don't have a shortage of such basic supplies. There is a regular supply from NGO called Ipas in collaborating with zonal health department” (man, age 40 years). The other key informant added, “Ipas supply us all necessary material for SAC service and no problem at all regarding basic supply” (woman, age 37 years).

The head of the zonal health department at MCH added, “We have medication for abortion, antibiotics, and anti-pain. Therefore, there is good basic supply from NGO called Ipas and Ethiopia drug supply agency. Ipas supply medication for abortion while Ethiopia drug supply agency supplies all according to request we send” (woman, 39 years), while another key informant added, “There are no medications, which are stocked out from the store for the past one year. Ipas constantly supplies medical abortion. Other medications are supplied with other health care service units by Ethiopia drug supply agency” (man, age 36 years).

One of the key informants on the availability of infrastructure said, “We have a shortage of room separated for SAC service. All SAC services (counselling and procedure) were provided in the delivery room. As result, it is not comfortable for clients to get SAC service in the delivery room as it exposed privacy issues. Since SAC service started in our health center, no room for SAC service separately till now. The other problem we have is electric light and water supply interruption, especially during summer time. Sometimes we appoint clients only for a temporary absence of light to confirm at least pregnancy diagnosis” (man, age 32 years).

In the study area, there were no shortages of infection prevention materials observed for the past year as the health facility received it consistently. One of the members of the zonal health office reported, “We have no shortage of infection prevention materials for the past one year. All health facilities are getting required materials as per their request from different suppliers including Ipas. Not only infection prevention materials, but also we have most of the resources required for these SAC services” (zonal health office member, age 39 years).

In the study area, all health facilities had baseline laboratory services required for the pre-SAC service, including complete blood count. A key informant from one health center said, “There are basic laboratory services which are needed in SAC service provision in our health center. Since I was assigned here, I did not come across an interruption of these laboratory service” (man, age 29 years).

Similarly, there was no shortage of manual vacuum aspirations (MVA) for surgical SAC procedures with a gestational age of less than 12 weeks despite its utilization. However, skilled staff who can operate the MVA were very limited. One of the key informants said, “There is no shortage of equipment including MVA for SAC surgical service despite of its update. Most of the time we do not use MVA for SAC service, instead we are using mifepristone and misoprostol because in health center only up to 12 weeks gestational age is allowed. Nevertheless, the equipment is too old. Additionally, we do not have trained professionals who can operate it for incomplete abortion. Hence I am not sure whether all are functional properly right now” (man, age 34 years).

Except for the procedural room and maternal waiting area, all [*n* = 7(100%)] health facilities have all the required materials and infrastructure resources according to the SAC guidelines. The resource availability was summarized to 94% and judged excellent (Table 2).

**Table 2:** Judgment matrix of resource availability indicators at North Shewa zone public health facilities as a national guideline.

Indicators	Expected (*a*)	Agreed (*b*)	Observed (*c*)	Achievements		Judgment parameter
				Value = (b×c)/a	%	
Proportion health facilities with basic equipment to provide SAC service	7 (100)	2.8	7	2.8	100	Excellent
Proportion health facilities with functional laboratory services available for SAC service	7 (100)	3.2	7	3.2	100	Excellent
Proportion of health facilities with basic supplies available to provide SAC service	7 (100)	3.5	7	3.5	100	Excellent
Proportion of health facilities with materials for infection prevention for SAC service	7 (100)	2.8	7	2.8	100	Excellent
Proportion of health facilities with equipment used for uterine evacuation as indicated by SAC guideline.	7 (100)	2.8	7	2.8	100	Excellent
Proportion of health facilities with trained staff available to provide service	7 (100)	3.5	7	3.5	100	Excellent
Proportion of health facilities with no stoke out of drugs for medical abortion in the past three month	7 (100)	3.5	7	3.5	100	Excellent
Proportion of health facilities with technical and procedural guideline	7 (100)	3.5	7	3.5	100	Excellent
Proportion of health facilities with CAC registration in each health facilities	7 (100)	2.5	7	2.5	100	Excellent
Proportion of health facilities with functional ambulance services for SAC service	7 (100)	1.8	7	1.8	100	Excellent
Proportion of health facilities with data recording formats in each health facilities	7 (100)	1.8	7	1.8	100	Excellent
Proportion of health facilities with procedural room for SAC clients	7 (100)	2.1	3	0.9	42	Poor
proportion of health facilities with maternal waiting area	7 (100)	1.4	3	0.6	43	Poor
Over all resources evaluation	*** ***	35		33	94	Excellent

### Compliance of healthcare workers with national SAC services

All healthcare workers’ ages fell in the intervals of 25–30 years (*n* = 14), 31–35 years (*n* = 32), and 36–40 years (*n* = 29). On the other hand, the experience of healthcare workers was in the range of 1–10 years. In total, 26 healthcare workers had 1–5 years of experience and 49 healthcare workers had 6–10 years of experience.

All clients were identified as HIV/AIDS target groups or not and were provided anti-pain medication during procedures as recommended by national guidelines. In addition, their HIV/AIDS status and their acceptance of the results were declared. Of the clients, 96% were provided counseling services on post-abortion family planning, while only three clients were encouraged to express their needs and desires during treatment. However, only nine (11.5%) healthcare workers gave their clients the opportunity to express their desires during interactions between clients and care providers. Of the care providers, 66 (88%) communicated in a language their clients understood and sent for baseline laboratory investigations. However, only nine care providers greeted their clients respectfully, and none of them provided information supported by information, education and communication (IEC) materials (Table 3).

**Table 3:** Number of healthcare providers who delivered SAC services at each health facility per the national guideline recommendations.

Activities	Health facilities									
	Yes/no	EHC	FGH	KGH	MTHC	DtsHC	YGHC	DDH	Total	%
Did she/he greeted respectfully?	Yes	0	5	4	0	0	0	0	9	12
Did she/he keep clients privacy	Yes	0	15	13	0	4	0	12	45	60
Did he/she communicated by language she understood by HCW	Yes	9	15	14	6	8	2	12	66	88
Did he/she take gynecological history?	Yes	8	9	11	8	9	2	8	57	76
Did he/she told the clients what procedure/steps going to provide to be provided	Yes	1	2	15	16	4	2	8	48	64
Did he/she did physical examination?	Yes	0	15	7	9	3	2	9	45	60
Did he/she provide information supported by IEC materials?	Yes	0	0	0	0	0	0	0	0	0
Did he/she take vital sign?	Yes	0	3	8	8	7	2	5	33	44
Did he/she send for laboratory service?	Yes	9	14	15	6	8	2	12	66	88
Did he/she worn infection prevention materials during surgical procedure	Yes	9	15	16	10	9	2	14	75	100
Did he/she provide anti pain?	Yes	9	15	16	10	9	2	14	75	100
Did he/she provide post abortion counseling?	Yes	8	15	16	8	7	2	14	72	96
Did he/she provide a chance for clients express her desire?	Yes	2	7	0	0	0	0	0	9	11.5
Did he/she provide contraceptive for clients?	Yes	8	14	11	9	7	8	12	69	92.3
Did he/she identify HIV/AIDS target?	Yes	9	15	16	10	9	2	14	75	100
Did he/she report clients’ HIV/AIDS test result?	Yes	9	15	16	10	9	2	14	75	100

The willingness to perform SAC services according to the criteria set by the national guidelines after they took the training varied from person to person. The majority were due to religious reasons while the rest were due to workload as SAC-trained human resources were very limited in the study area. One key informant emphasized, “There are health professionals who follow national guidelines in the SAC service profession and those who do not. The main reason for not following national guidelines was workload while unwillingness to perform was due to religion prohibition. Even some care providers did not register the clients and services provided to them. So, there may be services under-report” (woman, age 39 years). A facility head from one hospital added, “Care providers who took SAC training have work overload as they were also responsible for other maternal and child care services, and they are busy to do all national guideline recommendations. The other is that they feel tedious as they do it redundantly. Care providers who follow national guidelines pay attention to their profession. They do activities attentively by paying attention to what they do” (woman, age 40 years).

The overall compliance of healthcare workers with the safe abortion care service guidelines was summarized and found to be 62.3%, which was judged fair. Providing contraceptives, anti-pain medication, and identifying clients as HIV/AIDS test targets or not were areas where healthcare workers had excellent compliance, whereas explaining the steps going to be taken and performing physical examinations were areas where they had fair compliance (Table 4).

**Table 4:** Judgment of compliance indicators for SAC service in the health facilities of North Shewa zone.

Compliance indicators	Required (*a*)	Weight (*b*)	Observed (*c*)	Achievement		Parameters
				Value (*b *× *c*)/*ɑ*	%	
Proportion of care providers greeted clients respectfully	75 (100)	3	9	0.3	12	Poor
Proportion of care providers explained the steps of the procedure going to be done	75 (100)	2	48	1.28	64	Fair
Proportion of care providers who took gynecological history	75 (100)	1.99	57	1.44	72	Good
Proportion of care providers who did physical examination	75 (100)	2	45	1.2	60	Fair
Proportion of care providers who took vital sign before procedure	75 (100)	2.5	33	1	44	Poor
Proportion of care providers who provided contraceptive	75 (100)	3	69	2.8	96	Excellent
Proportion of care providers who provided anti pain	75 (100)	2	75	2	100	Excellent
Proportion of care providers who identified HIV/AIDS target or not	75 (100)	2	75	2	100	Excellent
Proportion of care providers who provided post abortion family planning counseling	75 (100)	1.67	72	1.6	96	Excellent
Proportion of care providers who declared clients’ HIV/AIDS test status	75 (100)	2	75	2	100	Excellent
Proportion of care providers who assisted their clients to express their desire	75 (100)	3	3	0.36	12	poor
Proportion of care providers who provided information supported by information education communication materials	75 (100)	2	0	0	0	Poor
Proportion of care providers who worn infection prevention materials	75 (100%)	2.5	25	2.5	100	Excellent
Proportion of care providers who sent for base line laboratory investigation	75 (100)	3	22	2.64	88	Excellent
Over all compliance evaluation		35		21.12	62.3	Fair

### Client exit interview (client satisfaction)

The clients had a mean age of 24.09 years. Of the 296 clients, 157 (53%) were aged 18–23 years; approximately half (53.7%) were unmarried and more than one-third (37.5%) had completed secondary school (grade 9–10). Moreover, 71.6% of the participants were followers of the Orthodox religion, 67.9% lived in urban areas, 47% of them were students, and 70.9% of current SAC services users were due to unwanted pregnancy (Table 5).

**Table 5:** Sociodemographic characteristics of respondents in the evaluation of SAC services at North Shewa zone health public health facilities (*n* = 296).

Variable	Categories	Frequency	%
Age	18–23	157	53.0
	24–29	111	37.5
	30–35	24	8.1
	36–40	4	1.4
Marital status	Married	92	31.1
	Single	159	53.7
	Separate	28	9.5
	Widowed	12	4.1
	Divorced	5	1.7
Educational status	Unable to read and write	4	1.4
	Those has no formal education	86	29.1
	Primary school (1–8)	60	20.3
	Secondary school (9–10)	111	37.5
	Preparatory school and above	35	11.8
Religion	Orthodox	212	71.6
	Protestant	70	23.6
	Wakefata	7	2.4
	Muslim	6	2
	Other	1	0.3
Ethnicity	Oromo	244	82.4
	Amhara	529	17.6
Residence	Urban	156	67.9
	Rural	140	32.1
Occupation	Student	139	47
	Employment	26	8.8
	Private	30	10.1
	Merchant	31	10.5
	Housewife	43	14.5
	Daily laborer	22	7.4
	Unemployment	5	1.7
Abortion history	Yes	10	3.4
	No	286	96.6
Reason of current abortion	Unwanted pregnancy	210	70.9
	Rape	29	9.8
	Health problem of mother	24	8.1
	Health problem of fetus	18	6.1
	Pregnancy from relative	5	1.7
	Physical and mental problem of mother	10	3.4

### Gynecological and service characteristics

Eight in 10 (81.1%) clients had one to two pregnancies (gravidity), most (90.5%) had 0–2 children (parity), over three-fourths (77%) had a gestational age of 4–12 weeks, and 77% received the SAC service within 1–3 days (79.4%). More than 6 in 10 (64.2%) clients received the SAC service from male care providers, while the majority 232 (78.4%) of the clients received a medical abortion (Table 6).

**Table 6:** Client- and service-related characteristics of respondents in the evaluation of SAC services at North Shewa zone public health facilities (*n* = 296).

Variable	Category	Frequency	%
Gravidity	1–2	225	76
	3–4	71	24
Parity	0–2	241	90.5
	3–5	55	9.5
Gestational age in week	4–12	228	77
	13–24	68	23
	Total	296	100
Duration it takes to receive SAC service	1–3 days	280	79.4
	4–6 days	16	20.6
Sex of care providers	Male	113	38
	Female	183	62
	Total	296	100
Type of uterine evacuation clients’ received	Medical abortion	232	78.4
	Surgical abortion (MVA)	64	21.6

### Level of client satisfaction

In total, 235 (79.4%), 229 (77.4%), and 220 (74.3%) clients were satisfied with the clearness of information given, cleanness of the abortion procedural room, and skill of care providers, respectively. A total of 165 (55.7%), 132 (44.6%), and 128 (43.2%) clients were dissatisfied with the respectfulness of care providers, privacy, and being given the opportunity to make decisions regarding their current problem respectfully (Table 7).

**Table 7:** Level of client satisfaction on each satisfaction measuring items for evaluation of SAC quality in North Shewa zone health facilities (*n* = 296).

Indicators	Very satisfied		Satisfied		Neither (neutral)		Dissatisfied		Very dissatisfied		Mean	SD
	No.	%	No.	%	No.	%	No.	%	No.	%		
Respectfulness of care provider	0	0	91	30.7	32	10.8	165	55.7	8	2.7	2.95	0.99
Privacy kept	1	0.3	142	48	21	7.1	128	43.2	4	1.4	2.99	0.99
How guard treats on entrance	10	3.4	177	59.8	72	24.3	35	11.8	2	0.7	3.46	0.82
Pre-procedural information given	13	4.4%	202	68.2	28	9.5	42	14.2	6	2	3.65	0.82
General information with post abortion complication	20	6.8	179	60.5	28	9.5	28	21.6	5	1.7	3.49	0.16
Skill of care providers	17	5.7	220	74.3	24	8.1	29	9.8	6	2	3.72	0.80
Pain control during procedure	6	2	213	72	19	6.4	49	16.6	9	3	3.63	0.82
Post abortion FP counselling	5	1.7	189	63.9	20	6.8	74	25	8	2.7	3.42	0.94
Duration it takes to receive SAC service	7	2.4	133	44.9	12	4.1	119	40.2	25	8.4	3.09	1.06
Cleanliness of abortion procedural room	1	0.3	229	77.4	19	6.4	38	12.8	9	3	3.64	0.75
Getting chance in decision making on their problem	3	1	172	58.1	14	4.7	100	33.8	7	2.4	3.30	0.98
General information given on RH	8	2.7	235	79.4	15	5.1	33	11.1	5	1.7	3.77	0.73
Easiness of getting laboratory	11	3.7	236	79.7	11	3.7	33	11.1	5	1.7	3.59	0.85

A key informant from one health center points it out as “As you know terminating the pregnancy is a sensitive issue and again it is a taboo and full of tension until get it terminating. Getting pregnant without getting married is taboo and sensitive in the community. Hence, being in any situation if they got SAC service they satisfied especially those with unnecessary pregnancy” (female midwife, age 38 years).

Of the study participants, 51% were satisfied with the waiting time. Approximately 62% of the study participants were satisfied with the counseling given on post-abortion complications and post-abortion family planning. The overall satisfaction of clients was measured as 65% (Table 8).

**Table 8:** Judgment of client satisfaction dimension and indicators of SAC service quality compared with judgmental criteria at North Shewa zone government health facilities.

Satisfaction indicators	*A* (%)	*B*	*C* (%)	Achievements		Parameter
				*D* (No)	*E* (%)	
Proportion of clients satisfied with easiness of getting laboratory service	100	10	86	9	90	Excellent
Proportion of clients satisfied to the pain management	100	5	67	3.33	66	Good
Proportion of clients satisfied with clarity of information given	100	5	70	3.5	70	Good
Proportion clients satisfied with two way communication during care providing	100	8	60	4.8	60	Fair
Proportion of clients satisfied with counseling provided on post abortion family planning	100	10	62	6.2	62	Fair
Proportion clients satisfied with respectfulness of care providers	100	10	49.6	7	70	Good
Proportion of clients satisfied with waiting time to receive the service	100	10	52.2	5.1	51	Fair
Proportion of clients who were satisfied to privacy of counseling room	100	10	51	7	51	Fair
Proportion of clients satisfied with the manner the guards treat them on entry	100	5	62	3.1	62	Fair
Proportion of clients satisfied with cleanliness of SAC services room	100	8	87	6.96	86	Excellent
Proportion of clients satisfied with technical skills of care providers	100	8	82	6.6	83	Very good
Proportion clients satisfied with information provided on safe abortion procedure going to be done	100	6	66.3	3.9	65	Good
Proportion of clients satisfied with information given on post abortion complication	100	5	62	3.1	62	Fair
Over all clients’ satisfaction	100	100		65	65	Good

Judgment parameter: ≥85% excellent, 75%–84% very good, 65%–74% good, 50%–64% fair, and <49% poor. *A* = expected weight; *B* = agreed weight; *C* = observed weight; *D* = finding in number (B×C/A); *E* = finding in percent (*D*/*C*) × 100%.

### Overall evaluation of SAC service

**Figure 2:**
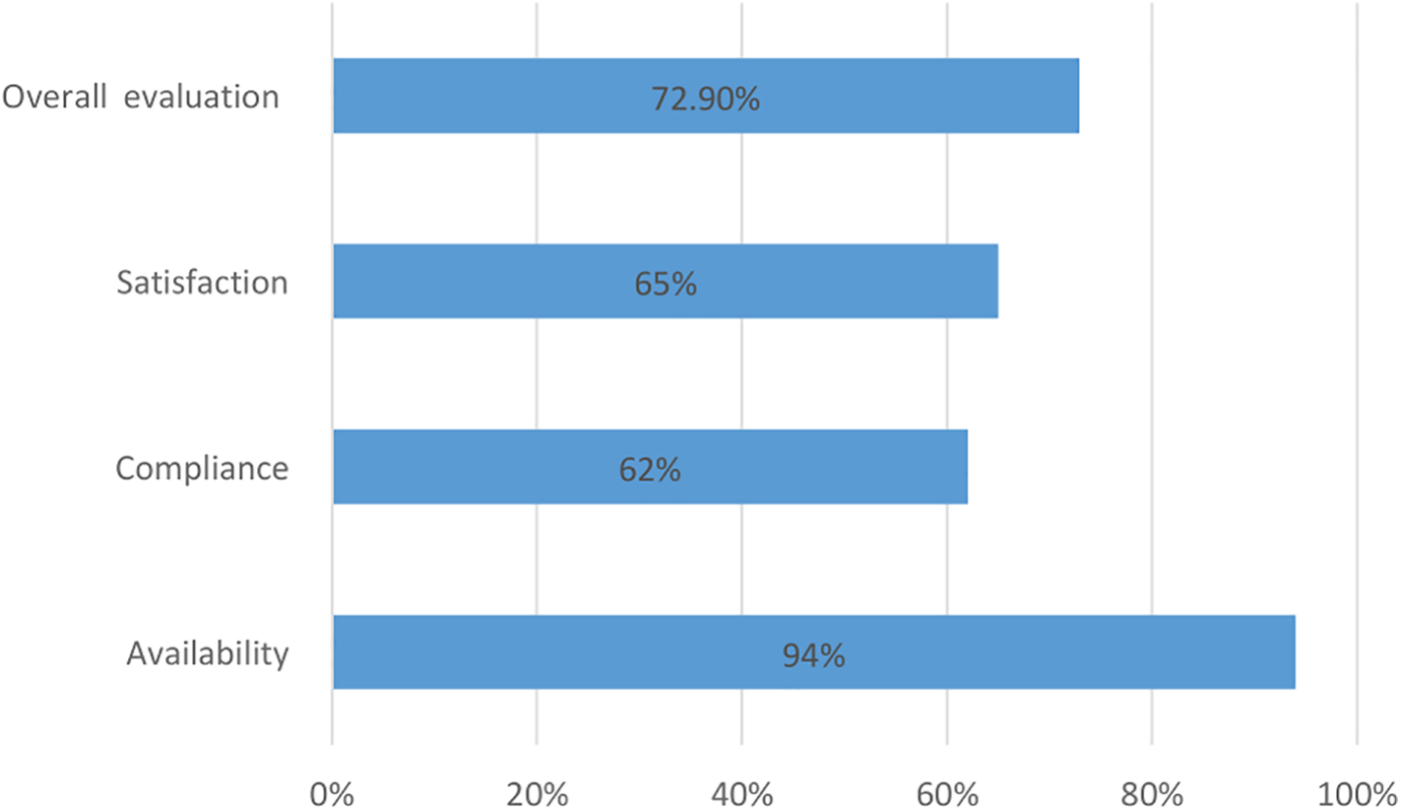
Summary report of resource availability, compliance, and overall SAC service evaluation at North Shewa zone.

The overall evaluation of SAC services was 72.9%, which was measured as resource availability (94%), healthcare worker compliance (60.3%), and client satisfaction (65%) (Figure 2).

### Factors associated with client satisfaction

Being a female healthcare worker led to an increment of 0.629 (95% CI = 0.569–0.716) in client satisfaction. Clients with a parity of <2 led to an increment of 0.361 (95% CI = 0.211–561) in satisfaction. However, clients residing in urban areas led to a reduction of 0.543 in satisfaction (95% CI = −0.472 to −0.3278) while clients aged 35–40 years led to a reduction of 0.040 in their satisfaction (95% CI = −0.077 to −0.004) (Table 9).

**Table 9:** Factors that affect client satisfaction toward SAC services at North Shewa zone public health facilities.

Variable	Frequency (%)	Unstandardized coefficient *B*	Standardized coefficient *B*	*p*-value	95% confidence interval for B
Constant		2.553		0.000	0.505 to 0.601
Sex of care providers (female)	183 (61.8)	0.629	0.767	<0.001**	0.569 to 0.716
Number of days taken to receive service (1–3 days)	280 (94.6)	0.019	0.052	0.368	−0.023 to 0.062
Residence (urban)	156 (67.9)	−0.543	−0.671	<0.001**	−0.472 to −0.327
Number of parity (0–2)	241 (81.4)	0.361	0.478	<0.001**	0.211 to 0.561
Clients’ age (30–35)	225 (76)	−0.040	−0.121	0.031*	−0.077 to −0.004
Gravidity (1–2)	225 (76)	0.010	0.051	0.369	−0.012 to 0.033

*Significant at *p*-value of 0.05.

**Significant at *p*-value of 0.01.

## Discussion

This evaluation was conducted to evaluate the SAC services with respect to resources, healthcare worker compliance, and client satisfaction. Accordingly, resource availability, healthcare worker compliance, and client satisfaction were 94%, 62.3%, and 65%, respectively.

Approximately three-fourths of HCW took SAC service training, which was comparable with a study carried out in Afghanistan where 70% of HCW took the training (22). However, it was higher than in a study carried out in other parts of Ethiopia and lower than that in a study in Togo (22, 23). The inconsistency may be due to the differences in study methodology, hospital policy, set up, and economic differences. An insufficient number of trained HCW on SAC service may lead to long waiting times for appointments, which may fatigue the clients. Moreover, they may practice unsafe abortion, which leads a rise in maternal mortality.

Only hospitals had obstetricians and none of the facilities had a non-obstetrician doctor who could serve in the SAC units. A similar study in India showed that 46% and 53.8% of health facilities had obstetricians and non-obstetricians, respectively, who could provide SAC services (24). In this case, our results were different from that study, which may be due to differences in health policy, economic characteristics, and study methodology. In the present study, most of the health facilities were health centers. In Ethiopia, specialized physicians still have not been assigned to rural health centers. Therefore, all complicated cases were referred to higher hospitals that employ specialized physicians. The absence of such physicians may contribute to maternal morbidity and mortality, especially among women residing in rural areas.

The results of this evaluation suggest that the number of HCW trained on SAC services was not adequate. Most key informants said that trained professionals on SAC were limited to midwives, which may result in the organizations providing training not doing it adequately. The inadequate number of trained HCW may lead to obstacles to providing service that may deteriorate the quality of SAC services. Nevertheless, a qualitative study conducted in Ghana showed that most hospital administrators and heads of departments who were not in favor of providing abortion services neither facilitate staff training nor procure MVA kits to enable skilled providers to offer safe abortion services. Some administrators were not even interested in discussing the topic or attending meetings where SAC service discussions are held. The reason given was that they hold positions in religious institutions and did not want to be seen to be associated with SAC services (25). Being a position holder in health organizations and in religious institutions at the same time in Ghana compromise the SAC services and increase the number of unsafe abortions, especially in low-resource settings.

The finding of this evaluation indicated that all the health facilities have the basic instruments that help to conduct SAC services, includes MVA, cannulas, connecting tubes, vacuum pumps with an extra tube, tenaculum, long sponge forceps, and clear glass dishes for tissue inspection. Similarly, all the health facilities had the necessary medications (mifepristone, misoprostol, diclofenac, ibuprofen, tramadol, ceftriaxone, metronidazole, and doxycycline) that were in line with WHO and national SAC service guideline recommendations (17, 18). However, in Nepal, only 32% of health facilities had essential instruments and abortion medications (26). A possible reason for the difference may be due to the difference in supply chain and budgets assigned for allocating these instruments. In the present study, most of the basic supplies, including abortion medication, were supplied by non-governmental organizations (Ipas) and the zonal health department on a regular basis.

This finding shows only four in seven (57%) health facilities had waiting areas, counseling rooms, and procedural rooms, which was lower than in the study from India (24). The possible variation may be due to differences in study methodology and economic differences between the two study areas. The absence of separate rooms for the SAC services deprived clients of both auditory and visual privacy, which further distracted clients from using the SAC service and leading them to undergo an unsafe abortion traditionally.

All the health facilities had basic supplies, including cotton, surgical gloves, disposable gloves, long needle holders, gauze, syringes, liger lactate, and normal saline, as recommended by WHO and national SAC guidelines (17, 18). The evaluation also identified that some of the studied health facilities had running water in the SAC service area. This result was lower than that of a study conducted in another region of Ethiopia, which showed that only hospitals had running water in the procedural room (22). This may be due to the difference in infrastructure distribution between the two study areas. The absence of water supply in SAC service units may be obstacles to infection prevention techniques during provision of the service.

In all the health facilities, infection prevention materials (eye goggles, masks, aprons, autoclaves, decontaminant solutions, storage of solid sharp material, and solid waste bins) were available. Moreover, most laboratory services needed for SAC services were available, including cervical cancer screening, venereal disease research laboratory test (VDRL), hemoglobin, urine analysis, blood group test, pregnancy test, Rh factor test, and HIV/AIDS test, as recommended by WHO and national SAC guidelines (17, 18). The availability of infection prevention materials leads to a decrease in infections, such as sepsis, especially in surgical abortion procedures. On the other hand, baseline laboratory services in the SAC unit ensures pregnancy diagnosis, effective SAC service, and identifies other medical cases that may be exacerbated by SAC procedures.

Our findings indicated that almost all care providers provided post-abortion family planning counseling. Similarly, a study from Zimbabwe showed that 94% of assessed clients were provided post-abortion family planning counseling (27), which was comparable with the findings in the present study. However, the current result was greater than in the study from other areas of Ethiopia, which revealed that only 75% of care providers provided post-abortion FP counseling (28). This inconsistency may be due to differences in study methods, trained care providers, and care provider commitment. Post-abortion FP is a fundamental intervention to prevent unwanted pregnancy, which would result in unsafe abortion. Therefore, improving the post-abortion FP service reduces recurrent abortions that may hurt women's future pregnancies (29). This study also shows that 23 (85.5%) care providers did not assist clients in expressing their desire for current needs, which is in line with a study from other areas (20, 28, 30).

According to this study, almost all care providers provided family planning counseling depending on clients’ preferences. This result is higher than in a study carried out in Nepal, which showed that 85% of care providers provided this service. Nevertheless, a study from Zimbabwe revealed only 46% of care providers provided FP advice (27, 31). This variation may be due to a contraceptive shortage, a lack of training, the unwillingness of clients to receive contraceptives, and non-compliant care providers in Zimbabwe. The current result was also congruent with WHO and national SAC service guidelines (2, 17).

In this study, almost no care providers showed respect to their clients during service provision. However, a similar study conducted in other settings in Ethiopia demonstrated that 93.5% of care providers show respect when providing SAC services (28). The variation may be a result of care providers’ attitudes toward pregnancy without being married, and care providers in other areas may be taking compassionate and respectful care (CRC) training. Respecting clients during service provision is a pivot to encourage clients to use modern treatments instead of traditional ones (32).

Three-fourths of care providers took the clients’ gynecological history before providing SAC services. This result was higher than those of a comparable study in Afghanistan that showed that only one-fourth of care providers took a gynecological history before starting SAC service provision (26). A possible justification for this variation may be due to the difference in study methodology, the scarcity of trained care providers, and the SAC service provision protocol. Taking clients’ gynecological history before SAC may lead to the right treatments and decrease complications that may arise from SAC service. This study shows that all the care workers had worn the necessary infection prevention material while providing SAC services. This result was similar to those of a study conducted in other Ethiopian regions, and the Tigray region shows all care providers wore infection prevention material during their interactions with clients (22, 33).

The findings of this evaluation indicated that the majority of the clients were not satisfied with HCW's respectfulness. However, a similar study conducted in Mexico City showed that most of the study participants were very satisfied with HCW's respectfulness (92% very satisfied) (20). This discrepancy may be due to the difference in attitude among care providers toward unwanted pregnancy and social taboo in the different setup. Whenever HCW's did not respect clients in the SAC service unit, the client may go for an unsafe traditional abortion, which may contribute to maternal mortality.

According to this evaluation, almost 4 in 50 clients were very dissatisfied with the time taken to receive SAC services after their first visit. This may lead to a decrease in willingness to seek care at health facilities. A comparable study from Ethiopia revealed that 5.3% of participants were very dissatisfied with the time spent in health facilities (16). Our study shows more clients were very dissatisfied compared to a study conducted in Jimma City. The variation could be due to the care providers’ efforts to make the client continue the pregnancy and a shortage of trained care providers. The other possible reason may be due to socioeconomic differences and the commitment of healthcare providers.

More than half of the clients were satisfied with the post-abortion family planning counseling. However, it was lower than that in a study conducted in Kyrgyzstan that showed 99% of clients were satisfied with the post-abortion FP counseling (34). The possible justification may be due to the difference in HCW's approach and the clients being able to participate in the services provided. The other reason may be the difference in study methodology and quality status.

Almost half the participants were satisfied with being able to express their desires during service provision, which may lead to rejecting the services provided to them and increase morbidity and maternal mortality. This result is far lower than that in a congruent study conducted in the Jimma zone, which showed that 82% were satisfied with being able to contribute to decision-making regarding their situation in the service provision (16). The variation may be due to the difference in person-centeredness care between the two zones.

In this evaluation study, almost three-fourths of the clients were satisfied with the practical skills of the care professionals. This finding was comparable to a study conducted in Jimma zone, in which 71.4% of the clients were satisfied with the skill of care providers (16). However, a study carried out in Mexico City revealed that 85% of participants were satisfied with the practical skills of the care providers (20). This variation may be due to differences in study methodology, quality of HCW training, and HCW's experience with SAC service provision.

The sex of care providers and a parity <2 were associated with client positivity. However, residing in urban areas and clients aged 30–35 years had a negative association. A study conducted in Mexico City also showed clients who had a parity <2 were less likely to attend SAC services (OR = 0.56, 95% CI = 0.33–0.95). A study from Tanzania indicated that clients with a parity <2 were more likely to be satisfied with SAC services (OR = 1.66, 95% CI = 1.10–2.52). A similarly study conducted in Nepal showed clients aged 25–35 years were less likely to be associated with this service (OR = 0.43, 95% CI = 0.19–0.97) (20, 26, 35). The variation in client satisfaction between our study and other studies may be because most clients in our study were unmarried and had a secondary school education. Giving birth before marriage is unacceptable in the wider community and terminating a pregnancy at a young age will affect future pregnancies.

## Conclusion

There were 75 health workers in maternal and child health departments in the studied health facilities. Except for the waiting area, interruption of water and electricity, and separate counseling and procedural rooms, all necessary resources were available. Healthcare providers were compliant with national guidelines in providing anti-pain medication, identifying clients as HIV/AIDS test targets or not, and declaring/reporting clients test results; however, they had poor compliance when respecting clients and checking vital signs before SAC procedures. Clients were fairly satisfied with waiting times, two-way communication, and post-abortion family planning counseling. The resources, compliance, and client satisfaction were measured and judged as excellent, fair, and good, respectively, while the overall evaluation was 72.9% and judged as good, which was below the expected result. All healthcare providers and other stakeholders should pay attention to provide services according to SAC service guideline recommendations to increase client satisfaction, which may increase utilization of SAC services.

## Data Availability

The raw data supporting the conclusions of this study are available from the corresponding author upon reasonable request.
