# Supplementary material for: Mixed-methods approach in evaluating safe abortion care services at public health facilities in North Shewa zone, central Ethiopia: a multicenter institutional cross-sectional study
Source: Front Health Serv. 2024 Jul 5;4:1352178. doi: 10.3389/frhs.2024.1352178 (PMC11258023; doi:10.3389/frhs.2024.1352178)
Supplement: Supplementary file 1 [file Table1.docx]

Supplementary Table 1: Material resources availability at the time study and for the past six months for SAC services at North shewa zone

| Basic supply | Availability of resources in each health facilities (yes/no) | | | | | | |
| --- | --- | --- | --- | --- | --- | --- | --- |
|  | FGH | KGH | DDH | EHC | MTHC | DtsHC | YGHC |
| Cotton | yes | yes | yes | yes | yes | yes | yes |
| Gauze | yes | yes | yes | yes | yes | yes | yes |
| Long needle holder | yes | yes | yes | yes | yes | yes | yes |
| Syringe | yes | yes | yes | yes | yes | yes | yes |
| Liger lactate | yes | yes | yes | yes | yes | yes | yes |
| Normal saline | yes | yes | yes | yes | yes | yes | yes |
| Disposable glove | yes | yes | yes | yes | yes | yes | ye |
| Surgical glove | yes | yes | yes | yes | yes | yes | yes |
| IEC material | yes | yes | yes | yes | yes | yes | yes |
| Misoprostol | yes | yes | yes | yes | yes | yes | yes |
| Mifepristone | yes | yes | yes | yes | yes | yes | yes |
| Diclofenac | yes | yes | yes | yes | yes | yes | yes |
| Ibuprofen | yes | yes | yes | yes | yes | yes | yes |
| Tramadol | yes | yes | yes | yes | yes | yes | yes |
| Ceftriaxone | yes | yes | yes | yes | yes | yes | yes |
| Metronidazole | yes | yes | yes | yes | yes | yes | yes |
| Doxycycline | yes | yes | yes | yes | yes | yes | yes |
| Presence of ambulance | Yes | Yes | Yes | Yes | Yes | Yes | Yes |
| *Presence of electric power | yes | yes | yes | Yes | Yes | yes | No |
| Presence of counseling room | Yes | Yes | Yes | No | No | Yes | No |
| Presence of examination table | Yes | Yes | Yes | Yes | Yes | Yes | Yes |
| Presence of procedural room | Yes | Yes | Yes | No | No | Yes | No |
| Presence of waiting area | Yes | Yes | Yes | No | No | Yes | No |
| *Presence of running water in procedural | Yes | Yes | Yes | Yes | Yes | Yes | Yes |
| Presence of functioning toilet | Yes | yes | yes | Yes | Yes | yes | Yes |
| Presence of eye goggle | yes | yes | Yes | yes | yes | Yes | yes |
| Presence mask | yes | yes | yes | yes | yes | Yes | yes |
| Functioning autoclave | yes | yes | Yes | yes | yes | Yes | yes |
| Apron | yes | yes | Yes | yes | yes | Yes | yes |
| Presence decontamination solution | yes | yes | Yes | yes | yes | Yes | yes |
| Presence of Storage of solid sharp material | yes | yes | Yes | yes | yes | Yes | yes |
| Presence solid waste bin | yes | yes | Yes | yes | yes | Yes | yes |
| presence of cervical cancer screening service | yes | yes | Yes | yes | yes | yes | yes |
| Presence of VDRL service test | yes | yes | yes | yes | yes | yes | yes |
| Presence of urine analysis service | yes | yes | Yes | yes | yes | yes | yes |
| Presence of HGB test | yes | yes | Yes | yes | yes | yes | yes |
| Presence of blood group test | yes | yes | Yes | yes | yes | yes | yes |
| Presence of pregnancy test | yes | yes | Yes | yes | yes | yes | yes |
| Presence HR factor test | yes | yes | Yes | yes | yes | yes | yes |
| Presence of HIV/AIDS test service | yes | yes | yes | yes | yes | yes | yes |
| Recording format | yes | yes | Yes | yes | yes | yes | yes |
| Reporting format | yes | yes | yes | yes | yes | yes | yes |
| CAC registration books | yes | yes | Yes | yes | yes | yes | yes |
| Presence of referral form | yes | yes | Yes | yes | yes | yes | yes |
| Presence consent form | yes | yes | Yes | yes | yes | yes | yes |
| Presence of national guideline | yes | yes | Yes | yes | yes | yes | yes |
| Vacuum aspirators | yes | yes | yes | yes | yes | yes | yes |
| Cannula | yes | yes | Yes | yes | yes | yes | yes |
| Connecting tube | yes | yes | Yes | yes | yes | yes | yes |
| Clear glass dish for tissue inspection | yes | yes | Yes | yes | yes | yes | yes |
| Vacuum pump with extra glass bottles | yes | yes | Yes | yes | yes | yes | yes |
| Single tooth tenaculum forceps | yes | yes | Yes | yes | yes | yes | yes |
| Container for cleansing solution | yes | yes | yes | yes | yes | yes | yes |
| Long sponge forceps | yes | yes | Yes | yes | yes | yes | yes |
| 7Plastic strainers | yes | yes | Yes | yes | yes | yes | yes |
| Local anesthesia 1% without adrenaline | yes | yes | Yes | yes | Yes | yes | yes |
| Pratt or Dennison dilators: size 13-27 French Medium self-retaining speculum | yes | yes | Yes | yes | Yes | yes | yes |
| Malleable metal sound | yes | yes | Yes | yes | Yes | yes | yes |
